# Case Report: Bullous Lung Disease Following COVID-19

**DOI:** 10.3389/fmed.2021.770778

**Published:** 2021-11-17

**Authors:** Prachi Pednekar, Kwesi Amoah, Robert Homer, Changwan Ryu, Denyse D. Lutchmansingh

**Affiliations:** ^1^Department of Internal Medicine, Bridgeport Hospital, Bridgeport, CT, United States; ^2^Section of Pulmonary, Critical Care, and Sleep Medicine, Bridgeport Hospital, Bridgeport, CT, United States; ^3^Department of Pathology, Yale School of Medicine, New Haven, CT, United States; ^4^Section of Pulmonary, Critical Care, and Sleep Medicine, Yale School of Medicine, New Haven, CT, United States

**Keywords:** COVID-19, bullous lung disease, post-acute COVID-19, SARS CoV-2, post-COVID “Long Haulers”

## Abstract

More than 87% of patients report the persistence of at least one symptom after recovery from the Coronavirus disease 2019 (COVID-19). Dyspnea is one of the most frequently reported symptoms following severe acute respiratory syndrome coronavirus-2 (SARS CoV-2) infection with persistent chest radiological abnormalities up to 3 months after symptom onset. These radiological abnormalities are variable and most commonly include ground-glass opacities, reticulations, mosaic attenuation, parenchymal bands, interlobular septal thickening, bronchiectasis, and fibrotic-like changes. However, in this case report, we describe findings of bullous lung disease as a complication of SARS CoV-2 infection. As the pandemic continues, there is a need to understand the multiple respiratory manifestations of post-acute sequelae of COVID-19. We, therefore, present this case to add to the current body of literature describing pulmonary disease as a consequence of SARS CoV-2 infection.

## Introduction

More than 87% of patients report at least one persistent symptom while recovering from Coronavirus Disease 2019 (COVID-19), with dyspnea being one of the most common following severe acute respiratory syndrome coronavirus-2 (SARS CoV-2) infection (1). Moreover, pulmonary function testing can demonstrate physiologic impairments, such as a reduction in diffusion capacity and restrictive ventilatory defects (2). Although the investigation into post-infectious pulmonary phenomena remains ongoing, interstitial lung disease has been the most frequently described, which has included findings of organizing pneumonia and fibrotic-like changes, such as reticulations, honeycombing, and traction bronchiectasis (3–5). In addition, small airways disease has been observed on chest imaging, namely, air-trapping and mosaic attenuation (5, 6). The presence of bullous lung disease has been infrequently reported, and in this case report, we describe this rare association post-COVID-19.

## Case

A 61-year-old gentleman without significant past medical history presented with worsening shortness of breath of 2 days duration. Three months prior to presentation, he was hospitalized for acute hypoxemic respiratory failure due to COVID-19 pneumonia managed by Bi-level Positive Airway Pressure, which was complicated by pneumomediastinum and right-sided pneumothorax that required chemical pleurodesis. This acute hospital course was also complicated by a left lower lobe pulmonary embolus for which he was anticoagulated with Apixaban. One week after discharge, he was re-admitted with increasing dyspnea. There was a concern for superimposed bacterial pneumonia but sputum cultures, blood cultures, and urine antigen testing for streptococcus pneumoniae and legionella were negative. He was treated with 10 days of antibiotics given the progression of respiratory symptoms and worsening hypoxia. Repeat CT angiogram showed progression of diffuse ground-glass opacities without evidence of pulmonary infarction with a new subsegmental pulmonary embolus in the right lower lobe. There was resolution of the previously noted left lower lobe pulmonary embolus with additional findings of a small pneumomediastinum. Ultrasound of the lower extremities was negative for deep vein thrombosis, and he was transitioned to Warfarin due to failure of anticoagulation with Apixaban.

**Figure 1:**
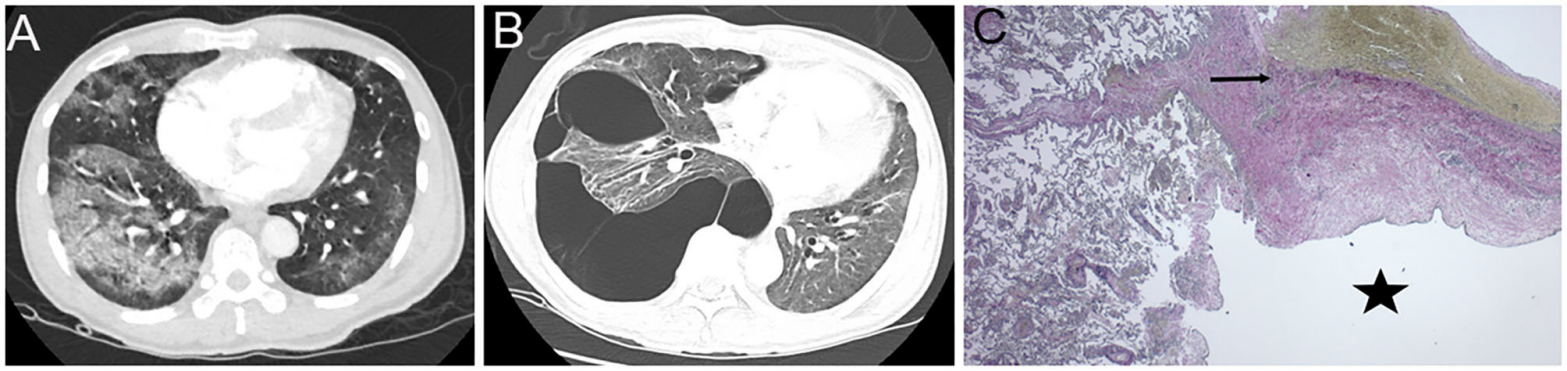
Representative CT images of the chest. **(A)** During acute COVID-19 infection, CT chest with intravenous (IV) contrast revealed diffuse ground glass with consolidative changes and no evidence of bullous lung disease (BLD). **(B)** Three-month post-infection, a repeat CT chest with IV contrast showed right-sided bullous lesions with mediastinal shift. **(C)** The right lower lobe lung section stained with Elastic Van Gieson (EVG) was used to identify the pleural elastic membrane (arrow) to confirm the presence of the bullae (star) localized within the lung parenchyma.

On this current presentation, he was hypoxemic, and a repeat CT scan of the chest showed extensive bullous lung disease throughout the right lung with mediastinal shift, not reported on prior imaging. He had no family history of lung disease or connective tissue disease. Prior to SARS CoV-2 infection, he was an avid marathon-runner, worked as a civil engineer with no occupational exposures, and never smoked tobacco products, vaped electronic cigarettes, or used illicit drugs. Other admission vitals were unremarkable, and a physical examination was only significant for decreased breath sounds on the right side. His laboratory data, such as repeat SARS CoV-2 nasal PCR, alpha-1 antitrypsin, and angiotensin-converting enzyme levels, were unremarkable. Cardiothoracic surgery was consulted, and he successfully underwent Video-Assisted Thoracoscopic surgery with wedge resection of the right lower lobe and chemical pleurodesis. Pathology ruled out a loculated pneumothorax and confirmed the presence of bullae localized within the lung parenchyma (Figure 1).

## Discussion

Bullous lung disease has been described secondary to cocaine, cigarette, or marijuana use and emphysema, sarcoidosis, alpha-1 antitrypsin deficiency, Marfan's syndrome, Ehlers-Danlos syndrome, and inhaled fiberglass exposure (7, 8). However, our patient did not have a family history of lung disease, occupational exposures, or a history of smoking, and his physical examination was not suggestive of either Marfan's or Ehlers-Danlos syndrome. As there was no evidence of underlying lung disease prior to his hospitalization, common etiologies of bullous lung disease were ruled out, and given the temporal association with SARS CoV-2 infection, we hypothesized that his bullous lung disease was related to COVID-19.

Up to 50% of survivors of SARS CoV-2 infection have residual radiological abnormalities more than 3 months after initial infection, similar to previous coronavirus infections, such as SARS and the Middle East respiratory syndrome (MERS) (5, 9–12). Older age, pre-existing co-morbidities, longer hospitalization, ICU admission, and a lower rate of steroid administration were more frequently associated with persistent imaging abnormalities (10). Commonly reported post-acute radiological sequelae include ground-glass opacities and reticulations, mosaic attenuation, parenchymal bands, interlobular septal thickening, bronchiectasis, and fibrotic-like changes (4, 5, 10). Additionally, some patients have been reported to have round cystic changes on CT, which might be associated with the process of resorption of consolidation potentially leading to the development of bullae (13). However, bullous lung disease has been less commonly described, and to date, only a few case reports have been noted in the literature (14–18).

Although the relationship between COVID-19 and the development of bullous lung disease is poorly understood, an emerging association has been described (19–21). Development of bullae following COVID-19 has been reported in male patients between 30 and 55 years of age with no prior history of pulmonary disease or history of mechanical ventilation, similar to our patient. The severity of acute presentation can vary between mild-to-moderate COVID-19 with bullous lung changes noted as early as 14 days after initial infection (15–18). Surgical intervention was not routinely offered to these patients and was only deemed necessary based on the presence of respiratory complications.

The mechanism of bleb formation and potential bullae development is unclear. Historically late-stage Acute Respiratory Distress Syndrome (ARDS) in patients on mechanical ventilation has been reported to be associated with a significantly higher number of bullous lesions and structural changes, with late-stage ARDS described as a restrictive lung disease with superimposed emphysema-like lesions (22). These findings support the hypothesis that structural changes over time may lead to an air-filled cavity kept open by a feeding airway acting as a one-way valve with subsequent formation of large lung bullae (22, 23). Multiple cases of delayed parenchymal destruction leading to bullous disease have been reported in the literature, which points toward some form of continued alveolar destructive process or lung parenchymal remodeling (16, 24, 25). Van der Klooster and Grootendorst have proposed that disruption of the protease–antiprotease balance in the lungs may be one of the underlying mechanisms related to cocaine or smoking-induced bullous lung disease, and there is evidence that this mechanism may exist in severe cases of pneumonia (26). Alternatively, Braun et al. reported a marked reduction in the functional activity of alpha-1 proteinase inhibitor in patients with acute pneumonia resulting in increased proteolytic damage of lung tissue (27). It may be possible that a similar imbalance occurs during SARS CoV-2 infection resulting in the formation of bullae.

Lung injury as a consequence of COVID-19 results in exudative diffuse alveolar damage (DAD) and later interstitial myofibroblastic proliferation and septal collagen deposition with mural fibrosis during the late or organizing stage of DAD (28). Sustained inflammation and alveolar injury may then result in further distension of alveoli with disruption of alveolar septa. These pathological changes are similar to prior lung injury noted during both the SARS and MERS epidemics where the development of fibrotic changes with septal thickening and formation of blebs were reported in infected patients (29, 30). These studies also showed the development of thin or thick reticular lines producing a lattice effect with significant distortion of lung anatomy and formation of organizing densities further into the disease course, likely predisposing to the formation of blebs (30). Similar findings of cystic lung disease in patients with a history of SARS without mechanical ventilation have also been reported (30). It was postulated that the formation of cysts and eventually blebs maybe associated with the development of ischemic parenchymal damage probably related to hypoxia, lung fibrosis, low lung compliance, and resultant injury from the inflammatory exudate (31–33). Whether this mechanism also occurs in patients who develop such lesions post-recovery from COVID-19 remains to be determined.

As the pandemic continues, there is a need to understand the multiple respiratory manifestations of post-acute sequelae of COVID-19. We present this case of bullous lung disease secondary to SARS CoV-2 infection to add to the literature with regard to post-COVID-19-related lung disease. While there has been concern centered around the potential development of interstitial lung disease, other respiratory complications do need to be identified. There is an imminent need for more investigative efforts to unravel the mechanisms leading to post-infectious lung injury and to identify risk factors for the development of post-infectious lung disease and determine the role of preventive therapy.

## Data Availability Statement

The original contributions presented in the study are included in the article/supplementary material, further inquiries can be directed to the corresponding author/s.

## Ethics Statement

Written informed consent was obtained from the individual(s) for the publication of any potentially identifiable images or data included in this article.

## Author Contributions

PP, KA, CR, and DL reviewed the medical literature, clinically managed the patient, prepared the figures, and manuscript. RH reviewed the relevant histopathology, prepared the figures, and manuscript. All the authors contributed to the conception, drafting, and final approval of the submitted work.

## Funding

This work was supported by the NIH/NHLBI 1K08HL151970-01 (CR).

## Conflict of Interest

The authors declare that the research was conducted in the absence of any commercial or financial relationships that could be construed as a potential conflict of interest.

## Publisher's Note

All claims expressed in this article are solely those of the authors and do not necessarily represent those of their affiliated organizations, or those of the publisher, the editors and the reviewers. Any product that may be evaluated in this article, or claim that may be made by its manufacturer, is not guaranteed or endorsed by the publisher.
